# The Use of Hyperscanning to Investigate the Role of Social, Affective, and Informative Gestures in Non-Verbal Communication. Electrophysiological (EEG) and Inter-Brain Connectivity Evidence

**DOI:** 10.3390/brainsci10010029

**Published:** 2020-01-05

**Authors:** Michela Balconi, Giulia Fronda

**Affiliations:** 1 Department of Psychology, Catholic University of the Sacred Heart, 20123 Milan, Italy; michela.balconi@unicatt.it; 2 Research Unit in Affective and Social Neuroscience, Catholic University of the Sacred Heart, 20123 Milan, Italy

**Keywords:** gestures, hyperscanning, inter-brain connectivity

## Abstract

Communication can be considered as a joint action that involves two or more individuals transmitting different information. In particular, non-verbal communication involves body movements used to communicate different information, characterized by the use of specific gestures. The present study aims to investigate the electrophysiological (EEG) correlates underlying the use of affective, social, and informative gestures during a non-verbal interaction between an encoder and decoder. From the results of the single brain and inter-brain analyses, an increase of frontal alpha, delta, and theta brain responsiveness and inter-brain connectivity emerged for affective and social gestures; while, for informative gestures, an increase of parietal alpha brain responsiveness and alpha, delta, and theta inter-brain connectivity was observed. Regarding the inter-agents’ role, an increase of frontal alpha activity was observed in the encoder compared to the decoder for social and affective gestures. Finally, regarding gesture valence, an increase of theta brain responsiveness and theta and beta inter-brain connectivity was observed for positive gestures on the left side compared to the right one. This study, therefore, revealed the function of the gesture type and valence in influencing individuals’ brain responsiveness and inter-brain connectivity, showing the presence of resonance mechanisms underlying gesture execution and observation.

## 1. Introduction

Communication is defined as a process that involves two or more individuals and allows the sharing of contents and information that can be transmitted in a verbal or non-verbal way.

Specifically, non-verbal communication has recently become more subject to investigation due to its considerable influence on the overall communication process and the social environment.

Non-verbal communication, in particular, consists of the transmission of information through the use of body movements and facial expressions, which can regulate, accentuate, and integrate the contents’ transmission. Several studies have observed bodily interaction within the communication process [1,2,3], recognizing gestures as the link between verbal and non-verbal communication [4,5], whose function has been investigated mainly within the interactional context [6,7,8,9,10]. 

In coordination with other units, gestures can create a broad action plan aimed at integrating, completing, and emphasizing communication [5]. Gestures consist of a group of motor actions intentionally directed towards an interlocutor to communicate something and regulate an individuals’ interactions. 

Considering the use of gestures in interactional contexts, neuroscience has been interested in exploring the cognitive and neural processes underlying the use of different gesture types. As shown by several studies, different brain areas appear to be involved in the observation and reproduction of gestures with different purposes [11,12,13]. Individually, the ventral and dorsal premotor cortex, the somatosensory areas, the anterior inferior parietal lobule [14], and the frontal cortex [15] appear to be primarily involved in the processes of gestures’ observation and reproduction. 

In particular, social gestures aim to regulate interactions with other individuals, starting, managing, or ending the relationship [16]. Affective gestures, instead, aim to influence the emotional state of another individual [17]. Informative gestures, finally, direct the attention of the decoder towards a specific object in the surrounding environment [18,19]. In addition to the typology of gestures, some studies have also investigated gesture valence by observing different brain responsiveness according to positive or negative connoted-gestures [20,21]. 

The frontal and parietal areas, specifically, are involved in mirroring processes, creating a direct link between gestures’ observation and execution [22,23], that allows for the understanding of the motor intention underlying others’ action reproductions and supports some cognitive, emotional, and empathic processes [24,25,26]. Moreover, frontal regions appear to be involved in mental model creation, including representations of the self and others [27,28,29,30].

To better investigate the neural processes and the mirroring mechanisms involved in a non-verbal communication exchange, the electrophysiological responses (EEG) of the encoder, who reproduces the gesture, and the decoder, who receives the gesture, have been recorded through the use of the hyperscanning paradigm during the reproduction of affective, social, and informative gestures. In particular, the use of hyperscanning, which consists of the simultaneous recording of the brain of two individuals involved in a common performance [31], has allowed for the investigation of non-verbal exchange as a social and interactional construct that cannot be traced back solely to the recording of the individual brain, which provides limited and incomplete information [32,33,34]. On the contrary, the use of hyperscanning allows the explanation of the complexity of non-verbal communication processes and permits us to observe the implicit coupling mechanisms occurring among inter-agent individuals [35,36].

Confirming the advantages of using hyperscanning, different studies have demonstrated its effectiveness in observing the mechanisms of brain synchronization present in the frontal cortex during cooperative exchanges [37,38], in the frontopolar cortex during verbal communication exchanges [39], and in the prefrontal cortex during empathic and prosocial behaviors [40,41].

This evidence shows how hyperscanning can provide valuable information on inter-brain connectivity, interpersonal coupling mechanisms, and social understanding processes [42].

Furthermore, the use of EEG to record individuals’ brain responses allowed for moment-by-moment recording of individuals’ interactions characterized by the reproduction of affective, social, and informative gestures [33,43].

In light of this evidence, we expected to observe a different neural response depending on the category and the valence of gestures reproduced. Specifically, we expected to observe an increase of low-frequency bands (delta and theta) and high-frequency bands (alpha and beta) activity in frontal areas during the reproduction of affective and social gestures. Indeed, according to the meaning of these gestures’ type, the frontal region is the one most implicated in social, relational, and emotional processes [44,45,46,47,48,49,50]. Instead, considering the meaning of informative gestures, we expected to observe a decrease of alpha activity in parietal areas due to an increase of attentional processes [51,52].

Considering also a gestures’ valence, as demonstrated by the model of neural signatures of affective experience [20,21], we expected to observe a more significant left-side brain response to positive gestures and a greater right-side brain response to negative ones.

Finally, considering the inter-agents’ role (encoder or decoder), we expected to observe an increase of alpha brain responsiveness (decrease of alpha power) in the encoder compared to in the decoder in the frontal areas for social and affective gestures, due to an increase of emotional response experienced during gesture reproduction [53,54] and to the association with previous experience [55,56,57].

Finally, considering inter-brain connectivity, we expected to observe an increase of inter-brain connectivity for the high and low-frequency bands in specific brain areas concerning gesture type and valence. Indeed, as demonstrated by different studies [29,58], during joint action, such as non-verbal communication, an increase of coordination and behavioral responses occurs between inter-agents’ individuals, creating implicit coupling mechanisms [59].

Specifically, about gesture type, we expected to observe an increase of alpha, delta, and theta inter-brain connectivity in the frontal areas for affective and social gestures and in the posterior areas for informative gestures. In particular, the activation of the frontal area can be related to its implication in relational, prosocial, and empathic processes [44,45,48,49], while the activation of parietal areas can be related to the involvement of processes concerning gestures’ observation and execution [60,61,62,63].

## 2. Materials and Methods

### 2.1. Subjects

Twenty-six participants coupled in dyads (M age = 23.45; SD age = 2.11) of the same gender were recruited for the experiment’s development. For the composition of the pairs, participants who were not familiar with each other were chosen. The roles of encoder and decoder were randomly assigned.

The following inclusion criteria were selected for the recruitment of participants: normal or corrected-to-normal visual acuity and right manual dominance. On the contrary, subjects under the age of 18 and above 40 were excluded from the research, as were those who presented clinical neurological disorders and who had experienced stressful life events in the previous 6 months. The research conduction was approved by the local ethics committee of the Department of Psychology of the Catholic University of the Sacred Heart (a.2017) and has followed the principles and guidelines of the Helsinki Declaration. The subjects were not paid for the research but gave their voluntary written consent to participate after signing the informed consent.

### 2.2. Procedure

The research required participants to sit facing each other with a computer located 60 cm away from both individuals to view the videos presented. Specifically, participants were asked to observe 60 videos reproducing different categories of gestures: social, affective, and informative of positive and negative valence that were administered through the use of the E-Prime 2.0 software (software E-prime2, Tools Psychology Software Inc., Sharpsburg, Pennsylvania, MD, USA).

In particular, videos reproduced a non-verbal exchange between two actors, one of which made a specific gesture towards another who received the gesture. The task required participants to reproduce the gesture seen in the video.

Specifically, one participant of the couple, randomly defined as the encoder, was asked to reproduce the gesture observed in the video to the partner, identified as the decoder, who was only asked to receive and comprehend the gesture. The task was administered in three different blocks so as not to tire the participants. 

**Figure 1:**
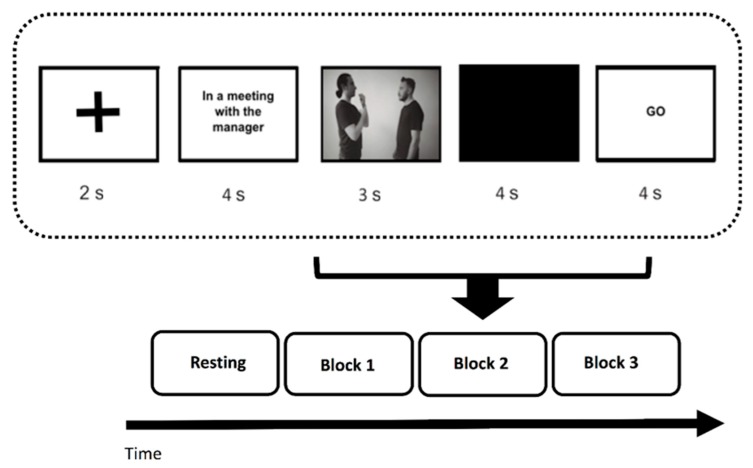
The figure shows the experimental procedure for the task administration.

Specifically, the following structure was used: the presentation of a 2 s blank screen, the presentation of a slide in which a context sentence was inserted to allow participants to better understand the meaning of the non-verbal communication exchange, the video presentation with the actors involved in the gestural communication, the inter-stimulus presentation lasting 4 s, and a “go” signal presentation to inform participants that they should have replayed the gesture. As mentioned above, 60 different videos were given: 10 reproducing positive social gestures, 10 reproducing negative social gestures, 10 reproducing positive affective gestures, 10 reproducing negative affective gestures, 10 reproducing positive informative gestures, and 10 reproducing negative informative gestures (Figure 1). 

Specifically, videos containing positive social gestures reproduce gestures that aim to start or maintain a relationship with the interlocutor; on the contrary, videos reproducing negative social gestures ask to reproduce gestures that have the purpose of interrupting the relationship with the interlocutor. Instead, the videos reproducing positive affective gestures reproduce gestures that have the purpose of communicating a state of psychological and physical well-being to the interlocutor contrary to those reproducing negative affective gestures that express a state of malaise.

Finally, for videos reproducing informative gestures, the valence is defined by the contest slide shown before the video.

Furthermore, 30 videos showed an interaction between two actors, both of male gender, while the other 30 videos reproduced an interaction between two female actresses. The stimuli used for the task were previously validated by 14 judges (M age = 28.34, SD age = 0.04) using a seven-point Likert scale according to the following categories: commonality, frequency of use, complexity, social meaning, familiarity, and emotional impact. The statistical analysis was used to define the categories of stimuli and verify the previous characteristics.

### 2.3. EEG Recording and Analysis

**Figure 2:**
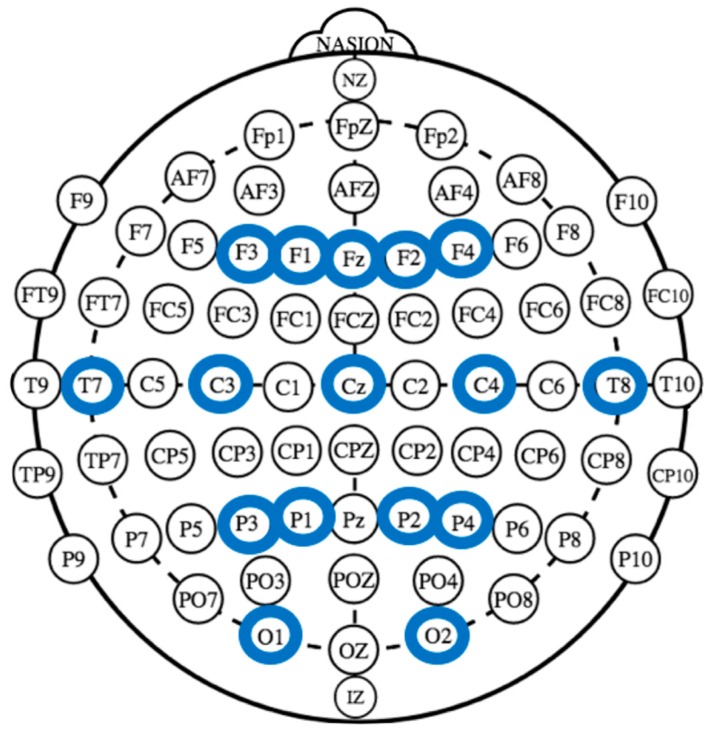
The figure shows the EEG channels location in the following positions: F3, F1, Fz, F2, F4, T7, C3, Cz, C4, T8, P3, P1, P2, P4, O1, and O2.

Two 16-channel EEG systems were used for the EEG signal recording (V-AMP: Brain Products, München; LiveAmp: Brain Products, GmbH, Gliching, Germany). Specifically, the electrodes were placed on the individual’s scalp with the use of two ElectroCaps at the following positions: F3, F1, Fz, F2, F4, T7, T8, C3, Cz, C4, P3, P1, P2, P4, O1, and O2 (Figure 2). Moreover, for the V-AMP system, an electrooculography (EOG) electrode has been positioned on the external canthi. A 5 kΩ electrode impedance was monitored before data collection for each individual. We used 1000 Hz for data sampling, with a 0.01–200 Hz input filter and a 50 Hz notch filter. Acquired data were filtered offline using a 0.5–40 Hz bandpass filter. Moreover, to reduce problems associated with signal-noise, a common offline average reference was calculated [64]. Concerning signal evaluation, portions of data containing artifacts were deleted, and an algorithm that uses a regression analysis in combination with the artifact average was utilized for ocular and motor artifacts correction. The EEG data were finally extracted in the frequency band: delta (0.5–4 Hz), theta (4–8 Hz), alpha (8–12 Hz), and beta (14–20 Hz) [65]. The mean EEG power for each channel and each frequency band was calculated by averaging data related only to the gesture reproduction phase using a 4-s segment. Finally, to obtain inter-brain connectivity, the partial correlation coefficient Πij was computed by normalizing the inverse of the covariance matrix: Γ = Σ − 1:

(1) $$\Pi ij=\frac{-\Gamma ij}{\sqrt{\Gamma ii\Gamma jj}}\;partial\;correlation\;matrix\;\Gamma \;=\;\left(\Gamma \;ij\;\right)=\;\Sigma \;-1\;inverse\;of\;the\;covariance\;matrix.$$

These measures represent the covariance of two signals, which allows the calculation of the partial correlation coefficients between two series of data in response to specific conditions (the experimental conditions).

## 3. Results

### 3.1. Data Analysis

Two sets of analyses were performed concerning EEG dependent measures. The first ANOVA applied on a single subject was considered for testing the effect of independent measures on each frequency band for each participant, independently from the dyad (single-brain analysis). The second set of analyses consisted of the inter-brain connectivity calculation for each band for each dyad. Since this was calculated for each pair of encoder/decoder it was finalized to compute the synchronization values within each couple for each measure. 

Then, we applied a second ANOVA to these inter-brain measures, to assess differences in synchrony strength across the experimental conditions (inter-brain connectivity analysis). 

For all the ANOVA tests, the degrees of freedom were corrected using Greenhouse–Geisser epsilon, where appropriate. Also, post-hoc comparisons (contrast analyses) were applied to the data.

The Bonferroni test was applied for multiple comparisons. In addition, the normality of the data distribution was preliminary tested (kurtosis and asymmetry tests). The normality assumption of the distribution was supported by these preliminary tests.

### 3.2. Single-Brain Analyses

For single-brain analyses, independent measures were: Role (encoder/decoder, 2), Valence (positive/negative, 2), Lateralization (left/right, 2), Gesture (social/ affective/informative, 3), and ROI (regions of interest, 4). Four ROI were calculated for left/right homologous sides for frontal (F3,F1-F2,F4), central (C3,C4), temporo-parietal (T7,P1-T8,P2) and occipital channels (O1,O2). 

A mixed model ANOVA was applied to the EEG bands.

#### 3.2.1. Alpha Band 

**Figure 3:**
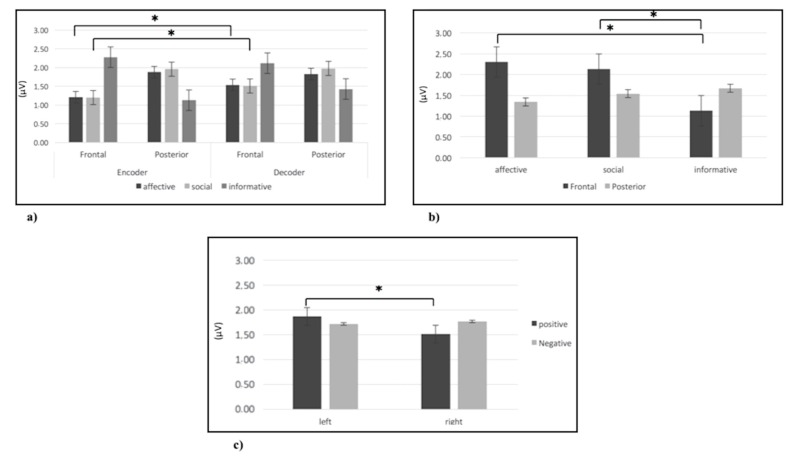
(**a**) Histogram of alpha brain activity for affective, social, and informative gestures in the frontal and posterior (temporo-parietal) areas in the encoder and decoder. The histogram shows an increase of brain activity (decrease of alpha power) in the frontal area for social and affective gestures in the encoder compared to the decoder. Bars represent ∓1SE. Stars mark statistically significant (*p <* 0.05) pairwise comparisons. (**b**) Histogram of delta brain activity for affective, social, and informative gestures in the frontal and posterior (temporo-parietal) areas. The histogram shows an increase of delta activity in the frontal area for affective and social gestures compared to informative gestures. Bars represent ∓1SE. Stars mark statistically significant (*p <* 0.05) pairwise comparisons. (**c**) Histogram of theta brain activity for positive and negative gestures in frontal left and right side. The histogram shows an increase of theta activity for positive gestures in the left side. Bars represent ∓1SE. Stars mark statistically significant (*p <* 0.05) pairwise comparisons.

Regarding alpha, as shown by ANOVA, a Role X Gesture X ROI significant interaction effect (F(6,152) = 9.13; *p <* 0.001; η2 = 0.36) was found. Specifically, post-hoc comparisons revealed an increase of brain activity (decrease of alpha power) in frontal area more than other areas (for all post-hoc comparisons *p* ≤ 0.001) for affective and social gestures compared to informative gestures and in posterior (temporo-parietal) area (for all post-hoc comparisons *p* ≤ 0.001) for informative gestures compared to affective and social gestures. Finally, concerning the inter-agents’ role, an increase of alpha activity (decrease of alpha power) in frontal area compared to others was observed for affective (F(1,24)= 9.78; *p <* 0.001; η2 = 0.37) and social gestures (F(1,24) = 10.09; *p <* 0.001; η2 = 0.39) in the encoder compared to the decoder (Figure 3a).

#### 3.2.2. Delta Band 

Regarding delta, as shown by ANOVA, a significant Gesture X ROI interaction effect was found (F(6,152) = 10.23; *p <* 0.001; η2 = 0.36). Specifically, post-hoc comparisons revealed an increase of delta activity in the frontal area compared to other areas for affective and social gestures compared to informative gestures (for all post-hoc comparisons *p* ≤ 0.001) (Figure 3b). 

#### 3.2.3. Theta Band

Regarding theta, as shown by ANOVA, a Valence X Lateralization X Gesture X ROI interaction effect was observed (F(6,152) = 10.13; *p <* 0.001; η2 = 0.38). Specifically, post-hoc comparisons revealed an increase of theta activity in the frontal area compared to other areas for affective and social gestures compared to informative gestures (for all post-hoc comparisons *p* ≤ 0.001). Moreover, an increase of theta activity was observed for positive gestures in the left frontal side compared to the right side (F(1,24) = 9.54; *p <* 0.001; η2 = 0.36) (Figure 3c). 

#### 3.2.4. Beta Band 

For the beta band, ANOVA reveals no significant effect.

### 3.3. Inter-Brain Connectivity Analyses

Starting from the raw database for each band, a second step was performed to calculate inter-subjects correlational indices finalized to compute the synchronization within each dyad. Such indices (correlation coefficients) were successively entered as dependent variables into mixed-model ANOVA tests, with Role, Valence, Lateralization, Gesture, and ROI as repeated factors. 

#### 3.3.1. Delta Band 

**Figure 4:**
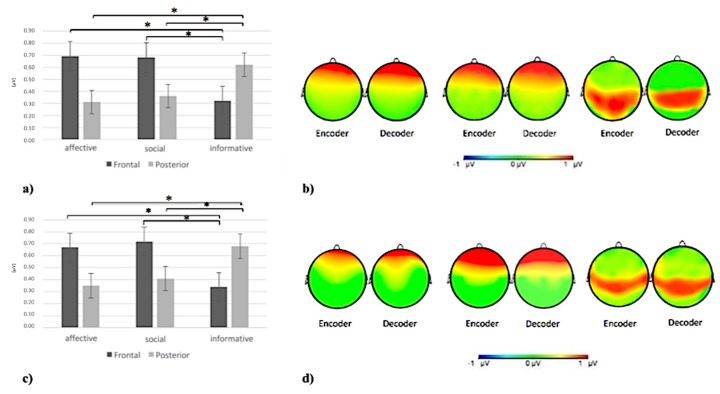
(**a**) Histogram of delta inter-brain connectivity for affective, social, and informative gestures in the frontal and posterior (temporo-parietal) areas. The histogram shows an increase of delta inter-brain connectivity in the frontal area for affective and social gestures and in the posterior (temporo-parietal) area for informative gestures. Bars represent ∓1SE. Stars mark statistically significant (*p <* 0.05) pairwise comparisons. (**b**) Delta inter-brain connectivity representation, from left to right, for affective, social, and informative gestures in the encoder and decoder. The red area represents the increase of delta inter-brain connectivity. (**c**) Histogram of alpha inter-brain connectivity for affective, social, and informative gestures in the frontal and posterior (temporo-parietal) areas. The histogram shows an increase of alpha inter-brain connectivity in the frontal area for affective and social gestures and in the temporo-parietal area for informative gestures. Bars represent ∓1SE. Stars mark statistically significant (*p <* 0.05) pairwise comparisons. (**d**) Alpha inter-brain connectivity representation, from left to right, for affective, social, and informative gestures in the encoder and decoder. The red area represents the increase of alpha inter-brain connectivity.

ANOVA revealed a significant Gesture X ROI interaction effect (F(6,152) = 8.45; *p <* 0.001; η2 = 0.33). Specifically, post-hoc comparisons revealed an increase of inter-brain connectivity in the frontal area more than other areas for affective and social gestures compared to informative ones and in posterior (temporo-parietal) area more than other areas for informative gestures (for all post-hoc comparisons *p* ≤ 0.001) compared to social and affective ones (Figure 4a,b). 

#### 3.3.2. Alpha Band 

As shown by ANOVA, a Gesture X ROI interaction effect was found (F(6,152) = 10.77; *p <* 0.001; η2 = 0.37). Specifically, post-hoc comparisons revealed an increase of inter-brain connectivity in the frontal area more than other areas for affective and social gestures compared to informative ones and in posterior area (temporo-parietal) more than other areas for informative gestures (for all post-hoc comparisons *p* ≤ 0.001) compared to affective and social ones (Figure 4c,d). 

#### 3.3.3. Theta Band 

**Figure 5:**
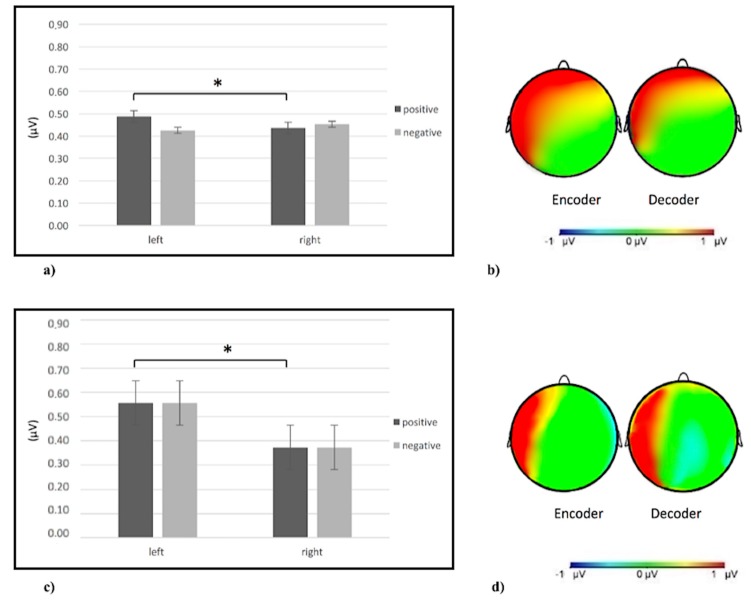
(**a**) Histogram of theta brain activity for positive and negative gestures in the left and right side. The figure shows an increase of theta power for positive gestures in the left side compared to the right one. Bars represent ∓1SE. Stars mark statistically significant (*p <* 0.05) pairwise comparisons. (**b**) Theta inter-brain connectivity representation, from left to right, for positive gestures in the encoder and decoder. The red area represents an increase of theta inter-brain connectivity. (**c**) Histogram of beta brain activity for positive and negative gestures for the left and right side. The figure shows an increase of beta power for positive gestures for the left side compared to the right one. Bars represent ∓1SE. Stars mark statistically significant (*p <* 0.05) pairwise comparisons. (**d**) Beta inter-brain connectivity representation, from left to right, for positive gestures in the encoder and decoder. The red area represents the increase of beta inter-brain connectivity.

As shown by ANOVA, a significant Valence X Lateralization X Gesture X ROI interaction effect was found (F(6,152) = 10.09; *p <* 0.001; η2 = 0.37). Specifically, post-hoc comparisons revealed an increase of inter-brain connectivity in the frontal area compared to others for affective and social gestures and in the posterior (temporo-parietal) area more than other areas for informative ones (for all post-hoc comparisons *p* ≤ 0.001). Furthermore, an increase of inter-brain connectivity for positive gestures has emerged in the left side compared to the right side (F(1,24) = 9.02; *p <* 0.001; η2 = 0.37) (Figure 5a,b). 

#### 3.3.4. Beta Band 

Regarding the beta band, as shown by ANOVA, a significant Valence X Lateralization interaction effect was found (F(6,152) = 9.55; *p <* 0.001; η2 = 0.35). Specifically ANOVA reveals an increase of inter-brain connectivity for positive gestures in the left side compare to the right one (F(1,24) = 9.95; *p <* 0.001; η2 = 0.38) (Figure 5c,d). 

## 4. Discussion

The present study set out to investigate the neural mechanisms underlying the reproduction of different gesture types (affective, social, and informative) with positive and negative valence. In particular, individuals’ brain responsiveness and neural inter-brain synchronization between the encoder, who reproduced the gesture, and the decoder, who received the gesture, were observed.

Compared to our initial hypotheses, we expected to observe different individuals’ brain responsiveness according to gesture type and valence. Furthermore, considering the inter-agents’ role, we expected to find a decrease of alpha power (increase of alpha brain responsiveness) in the frontal areas for affective and social gesture in the encoder compared to the decoder. 

Following the first hypothesis, a decrease of alpha power (i.e., an increase of brain responsiveness) was observed in frontal regions for social and affective gestures compared to informative ones. The increase of alpha brain responsiveness in the frontal areas may be due to the implementation of somatosensory and visuospatial processes used during the reproduction and reception of affective and social gestures [51,66,67]. Furthermore, a decrease of alpha power (an increase of alpha brain responsiveness) was observed in the posterior areas (temporo-parietal) for informative gestures concerning the implementation of more specifically visuospatial and attentional mechanisms [52,68] required by this type of gestures. 

Moreover, considering the inter-agents’ role, according to our starting hypothesis, an increase of alpha frontal brain responsiveness for social and affective gestures was observed in the encoder, compared to the decoder. 

The increase of alpha brain responsiveness in the encoder may be because the alpha band is sensitive to previous experience with actions, which lead the encoder to associate gestures reproduced with his previous experiences and contexts of use [55,56,57].

Furthermore, this result could also be associated with an increase in emotional response experienced by the encoder during the gesture’s reproduction. 

As demonstrated by different studies, an increase of alpha brain responsiveness occurs during the testing of behavioral arousal states, active engagement, and emotional excitement [53,54].

In addition to the alpha band, for affective and social gestures, an increase of frontal delta and theta activity has emerged. Interpreting this result in the light of the implicit and explicit meaning of affective and social gestures, aiming to influence the emotional state of the interlocutor and manage the social relationship, the greater response of these frequency bands in the frontal area can be related to the involvement of social, affective, relational, and empathic processes [20,69]. This interpretation is also supported by several studies that have observed an increase of delta and theta frontal activity in response to socio-emotional situations that involved emotional processes [44,46,48,49,50]. 

In addition, the present study also aimed to observe the neural effects of gesture valence, observing an increase of theta frontal left-side activity compared to the right one for positive gestures.

This result appears in agreement with our initial hypothesis, confirming the theory of neural signatures of affective experience [20,21,70], which postulates an increase of left-brain responsiveness according to positive stimuli, inducing an “approaching behavior”, compared to negative ones, which provides an increase of right-brain responsiveness inducing an “avoidance behavior” [71]. 

In addition to single-brain analysis, the present study also considered inter-agents’ inter-brain connectivity in order to investigate individuals’ resonance mechanisms and implicit neural coupling.

In this regard, compared to our starting hypotheses, we expected to observe a different individuals’ inter-brain connectivity according to gesture type and valence. Moreover, regarding inter-agents’ roles, we expected to observe an increase of inter-brain connectivity in both individuals (encoders and decoders) in specific brain areas concerning gesture type and valence.

In accordance with our starting hypothesis, from inter-brain connectivity, an increase of alpha, delta, and theta inter-brain connectivity has emerged in the frontal areas for affective and social gestures and in the posterior (temporo-parietal) areas for informative ones. 

The increase of inter-brain connectivity in these brain areas underlines the presence of mirroring mechanisms that are involved in gesture perception and execution [24].

Moreover, this result confirms the involvement of the fronto-parietal circuit in mirroring processes that provide a direct coupling between action observation and execution [72,73,74], leading to the activation of the same brain areas in both individuals involved in the exchange [57,75]. 

As demonstrated by previous studies, indeed, the development of common activities leads the individuals involved in the exchange to automatically align their behavior on different levels [76], and this led to the implementation of reciprocal modeling and interpersonal coupling mechanisms [37,77,78]. Specifically, the increase of frontal and posterior alpha inter-brain connectivity both in the encoder and decoder may be due to simultaneously generalized and joined attentional mechanisms present during the reproduction and comprehension of the gesture [51,66].

On the contrary, the increase of frontal delta and theta inter-brain connectivity in relation to affective and social gestures may indicate an increase of individuals’ emotional attunement provided by the involvement of emotional and empathic processing [44,45,46,50], while the increase of temporo-parietal delta and theta inter-brain connectivity for informative gestures can be due to perceptual processes involved in gesture observation and execution [22,23].

Finally, an increase of inter-brain connectivity was also observed for theta and beta bands according to gesture valence (positive or negative). In particular, an increase of theta and beta inter-brain connectivity was observed for positive gestures on the left-brain side compared to the right one.

This result confirms the presence of resonance and mirroring mechanisms in the encoder and decoder in correspondence with positive gestures that have the purpose of starting and maintaining a relationship with the interlocutor. Furthermore, the increase of theta inter-brain connectivity in relation to positive gestures may be due to the involvement of emotional mechanisms [79,80,81] required by positive gestures. Instead, the increase of beta inter-brain connectivity may be due, as shown by previous studies, to mechanisms of awareness, intentionality, and action planning [82] experienced during positive gestures reproduction and reception. 

To summarize, the results of inter-brain connectivity analyses reveal the presence of implicit mirroring and coupling mechanisms in both the encoder and decoder according to gesture type and valence. Finally, this evidence underlines the validity of hyperscanning as a paradigm that is useful for the investigation of the implicit neural mechanisms that take place in specific brain areas during common action performance, thus allowing one to observe the synergy and attunement mechanisms between the individuals involved in the exchange.

## 5. Conclusions

In conclusion, the results of the study have underlined how the meaning of the use of different types of gestures, such as social, affective, and informative ones, provide different individual neural activations, emphasizing an increase of frontal alpha, delta, and theta brain responsiveness and inter-brain connectivity for affective and social gestures. Moreover, an increase of parietal alpha brain responsiveness and alpha, delta, and theta inter-brain connectivity was observed for informative gestures. Furthermore, in relation to the role of inter-agents’ individuals, an increase of frontal alpha brain responsiveness has been observed in the encoder compared to the decoder for social and affective gestures. Instead, regarding inter-brain connectivity, an increase of alpha, delta, and theta inter-brain connectivity was observed in both the encoder and decoder in specific areas according to gesture type and valence. Taken together, this result underlines the presence of resonance and implicit coupling mechanisms that occur between individuals involved in the non-verbal exchange.

This study has, therefore, provided an overview of the functionality of specific types of gestures within a non-verbal interaction between the encoder and decoder. Furthermore, the present study has underlined the potentiality and validity of the hyperscanning technique in providing valuable information on inter-brain connectivity, interpersonal coupling mechanisms, and social understanding processes.

Despite the potentiality and originality of this study, it is not exempt from limitations. For example, a larger sample size could have been implemented to provide further evidence.

Furthermore, the use of other detection or neuroimaging methodologies would have allowed the integration of new measures, which would have then provided further supporting data. In addition, due to the relevance of autonomic system measures, future research should also include these data to better explore the relationship between gesture representation and autonomic markers, such as skin conductivity or heart rate variability.
